# Study on the Rationality for Antiviral Activity of *Flos Lonicerae Japonicae*-Fructus Forsythiae Herb Couple Preparations Improved by Chito-Oligosaccharide via Integral Pharmacokinetics

**DOI:** 10.3390/molecules22040654

**Published:** 2017-04-20

**Authors:** Wei Zhou, Ailing Yin, Jinjun Shan, Shouchuan Wang, Baochang Cai, Liuqing Di

**Affiliations:** 1 College of Pharmacy, Nanjing University of Chinese Medicine, Nanjing 210023, China; zhouwei19860506@163.com (W.Z.); bccai@126.com (B.C.); 2 Jiangsu Engineering Research Center for Efficient Delivery System of TCM, Nanjing 210023, China; 3 Nanjing Engineering Research Center for Industrialization of Chinese Medicine Pellets, Nanjing 210023, China; 4 Faculty of Health Sciences, University of Macau, Macau SAR, China; 5 Department of Pharmacy, Third Affiliated Hospital of Nanjing University of Chinese Medicine, Nanjing 210023, China; yal120_120@163.com; 6 Jiangsu Key Laboratory of Pediatric Respiratory Disease, Institute of Paediatrics, Nanjing University of Chinese Medicine, Nanjing 210021, China; dfsjj@163.com (J.S.); wscnj@126.com (S.W.)

**Keywords:** *Flos Lonicerae Japonicae*-Fructus forsythiae herb couple, integral pharmacokinetics, multiple caffeic acid derivatives, chito-oligosaccharide, tight junction, anti-viral activity

## Abstract

In the present study, the rationality for the antiviral effect (H1N1 virus) of *Flos Lonicerae Japonicae* (FLJ, named JinYinHua)-Fructus forsythiae (FF, named LianQiao) herb couple preparations improved by chito-oligosaccharide (COS) was investigated. We found that the improvement of antiviral activity for four preparations attributed to the enhancement of bioavailability for the FLJ-FF herb couple in vivo, and that caffeic acid derivatives are the most important type of components for antiviral activity. The anti-Influenza virus activity-half maximal inhibitory concentration (IC_50_), not area under concentration (AUC) was considered as the weighting factor for integrating the pharmacokinetics of caffeic acid derivatives. It was found that the integral absorption, both in vitro and in vivo, especially that in Shuang-Huang-Lian, can be improved significantly by COS, an absorption enhancer based on tight junction. The results indicated that the antiviral activity in four preparations improved by COS was mainly attributed to the integral absorption enhancement of caffeic acid derivatives.

## 1. Introduction

**Figure 1:**
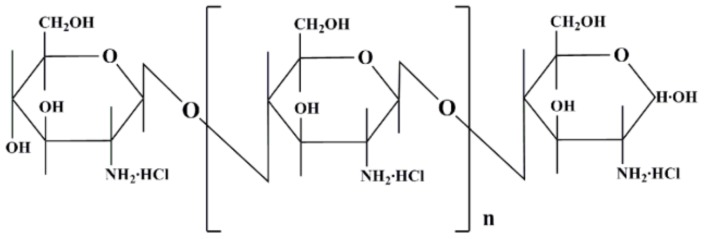
Chemical structure of COS.

*Flos Lonicerae Japonicae* (FLJ, named JinYinHua) possesses wide pharmacological actions, such as antivirus, antibacterial, anti-inflammation, antiendotoxin, blood fat reduction, etc. [1] and *Fructus Forsythiae* (FF, named LianQiao) has antiviral, antibacterial, antioxidant, anti-inflammatory effects, etc. [2]. The two herbs together (the ratio of FLJ:FF usually reached 1:1 showed maximum pharmacological effects [3]) in couples, named Yaodui in China, are the basic components of Chinese herbal preparations, such as Shuang-Huang-Lian oral liquid, Qin-Re-Jie-Du oral liquid, Fufang Qin-Lan oral liquid, and Yin-Qiao-Jie-Du tablet, which are used for treating acute upper respiratory tract infections by influenza [4,5,6,7,8,9,10]. Previously, we found that the chito-oligosaccharide (COS) (Figure 1), a promising excipient for absorption enhancement based on tight junctions of gastrointestinal drug [11], could enhance intestinal absorption of forsythoside A and chlorogenic acid, both in vitro and in vivo, and improve the antiviral activity (H1N1 virus) of FLJ-FF herb couple preparations significantly via in vitro influenza virus propagated in MDCK cells, but few studies have illustrated why the antiviral effect (H1N1 virus) can be improved by COS [12]. It was presumed that the bioavailability of FLJ-FF herb couple in preparations improved by COS resulted in their pharmacological effect enhancement.

**Figure 2:**
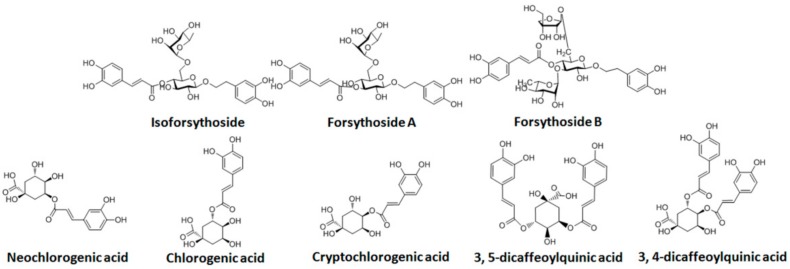
Chemical structure of caffeic acid derivatives.

It was reported [13,14,15,16] that the FLJ-FF herb couple contained flavones, isoflavones, phenolic acids, iridoids, phenylethanoid glycosides, lignans, and a few saponins. Caffeic acid derivatives (Figure 2) (isoforsythoside, forsythoside A, forsythoside B, neochlorogenic acid, chlorogenic acid, cryptochlorogenic acid, 3,5-dicaffeoylquinic acid, and 3,4-dicaffeoylquinic acid), possessing strong antiviral, antibacterial, and antioxidant activities, in vitro and in vivo [17,18,19,20,21,22], had a good positive correlation between dose and antiviral activity via both the pharmacokinetic/pharmacodynamic (PK/PD) model combined with the partial least-squares (PLS) method [18] and drug-drug interaction (DDI) combined with hierarchical cluster analysis (HCA) and principal component analysis (PCA) [23], and the antiviral activity was improved as the contents of caffeic acid derivatives in FLJ-FF herb couple enhanced, showing a remarkable positive correlation. As illustrated above, the caffeic acid derivatives were the main ingredients to control the quality of the FLJ-FF herb couple. It was presumed further that the bioavailability of caffeic acid derivatives in the FLJ-FF herb couple improved by COS could result in the antiviral activity enhancement in four preparations.

Owning to various caffeic acid derivatives with different antiviral efficacies, it is necessary to establish an integral pharmacokinetic approach to evaluate their contribution to overall effect. In the previous study, an area under concentration (AUC)-weighting method based on same type of constituents derived from traditional Chinese medicine (TCM) herbs has been created to obtain the holistic pharmacokinetic properties [24,25,26,27,28]. However, we found that the AUC value of chlorogenic acids (neochlorogenic acid, chlorogenic acid, and cryptochlorogenic acid) in the caffeic acid derivatives were largely higher than that of phenylethanoid glycosides (isoforsythoside, forsythoside A, and forsythoside B), but their antiviral activity was opposite. It was presumed that an efficacy-weighting approach might be more suitable for studying integral pharmacokinetics of caffeic acid derivatives than an AUC-weighting method.

Therefore, the current studies were mainly focused on: (1) Confirming whether the pharmacological effect enhancement in preparations is mainly due to the bioavailability of the FLJ-FF herb couple; (2) Studying whether the integral pharmacokinetics of caffeic acid derivatives calculated by the efficacy-weighting approach was more suitable than that obtained by the AUC-weighting method; (3) Investigating whether the integral absorption of caffeic acid derivatives in preparations of both in vitro and in vivo can be improved significantly by COS.

## 2. Results

### 2.1. Study on the Presumption that the Antiviral Effect Enhancement in the Preparations Resulted from the Bioavailability of the FLJ-FF Herb Couple Improved by COS

MTT test showed that 10% drug-containing serum in different time points had little effect on the cell viability.

**Figure 3:**
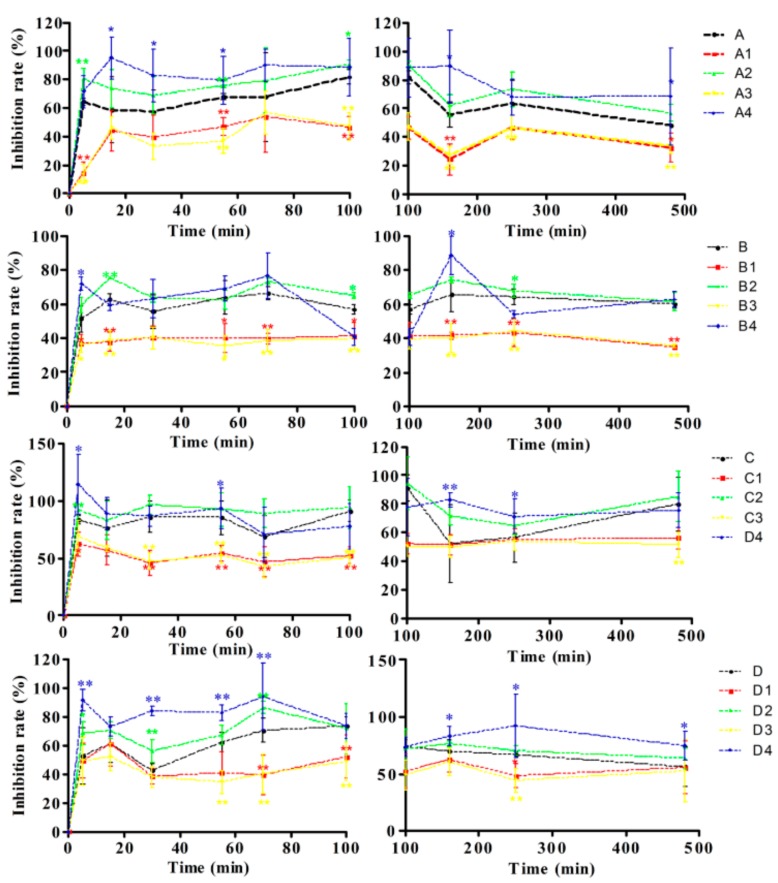
Effect of Shuang-Huang-Lian groups (A, A1, A2, A3 and A4), Yin-Qiao-Jie-Du groups (B, B1, B2, B3 and B4), Fufang Qin-Lan groups (C, C1, C2, C3 and C4) and Qin-Re-Jie-Du groups (D, D1, D2, D3 and D4) on influenza virus. (*) *p* < 0.05 and (**) *p* < 0.01, compared with the A, B, C and D groups, respectively (Mean ± SD, *n* = 6).

As shown in Figure S1, the antiviral model was built successfully. The inhibition rate of the COS group was not significant compared with that of the PBS group, although there was a remarkable increase in the inhibition rate value after administrating orally 20 mg/kg ribavirin as a positive control. Surprisingly, compared with that in control groups (A, B, C and D) (Figure 3), the antiviral effect was decreased significantly in the A1, B2, C1 and D1 group, but increased significantly in the A2, B2, C2 and D2 groups. Besides, the antiviral activity in the A3, B3, C3 and D3 groups cannot be enhanced by COS, but improved significantly in A4, B4, C4 and D4 groups. The results indicated that the pharmacological effect improved by COS was dependent on the bioavailability of the FLJ-FF herb couple enhancement.

### 2.2. Study on the Rationality for Integral Pharmacokinetics of Caffeic Acid Derivatives Calculated by the Efficacy-Weighting Approach and the AUC-Weighting Method

**Figure 4:**
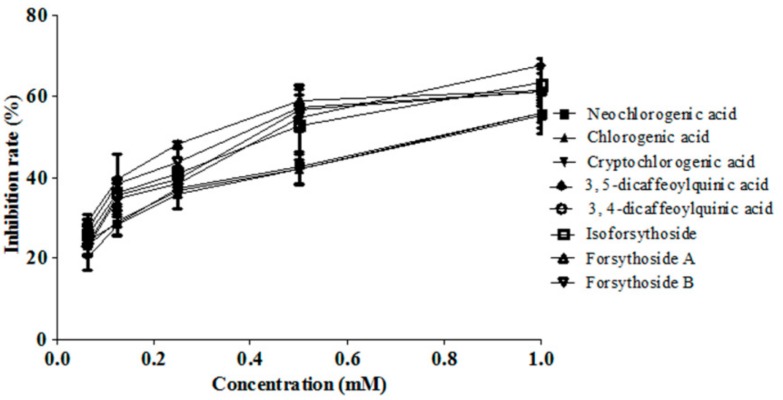
Effect of caffeic acid derivatives of different concentrations on the influenza virus. Inhibition rate was assayed with MTT and expressed as a percentage of controls (Mean ± SD, *n* = 8).

**Figure 5:**
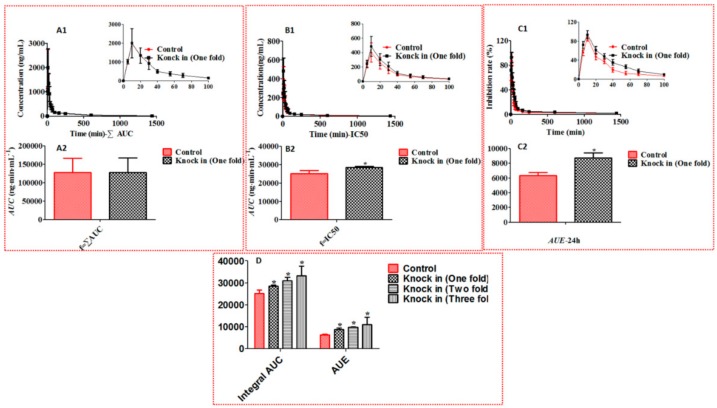
Integral pharmacokinetic parameters (AUC_0–24h_) and antiviral efficacy (AUE_0–24h_) of caffeic acid derivatives in the FLJ-FF herb couple knocked or without knocked in forsythoside A. (*) *p* < 0.05 compared with the control group. (**A1**,**A2**) represent integral mean pharmacokinetic profiles and AUC_0–24h_ based on AUC, respectively; (**B1**,**B2**) represent integral mean pharmacokinetic profiles and AUC_0–24h_ based on IC_50_, respectively; (**C1**,**C2**) represent mean inhibition profiles and AUE_0–2__4h_, respectively; (**D**) represents the correlation between integral AUC_0–24h_ based on IC_50_ and *A*UE_0–24h_ after the concentration of forsythoside A gradually knocked in the FLJ-FF herb couple (Mean ± SD, *n* = 6).

As shown in Figure 4 and Table 1, the rank order of antiviral activity for caffeic acid derivatives was forsythoside A > forsythoside B > isoforsythoside ≈ 3,4-dicaffeoylquinic acid ≈ 3,5-dicaffeoylquinic acid > neochlorogenic acid ≈ chlorogenic acid ≈ cryptochlorogenic acid. The weighting factors based on IC_50_ was shown in Table 1, the weighting factor of phenylethanoid glycosides was higher significantly that that of chlorogenic acids. For example, the weighting factor of forsythoside A was 2.47 times higher than that of chlorogenic acid. However, the weighting factor based on AUC of phenylethanoid glycosides was lower significantly than that of chlorogenic acids (Table 2). For example, the weighting factor of forsythoside A was 42.3 times lower than that of chlorogenic acid. In order to judge which weight coefficient was more suitable for integral pharmacokinetics of caffeic acid derivatives, we compared both the integral AUC and antiviral effect in the FLJ-FF herb couple as the control group with that in the FLJ-FF herb couple knocked in forsythoside A as the experimental group. Figure 5C showed that the antiviral effect in the experimental group was significantly better than that in the control group, and the integral AUC calculated by IC_50_ (Figure 5B), not AUC (Figure 5A)as weight coefficient index was also improved significantly, and Figure 5D showed that the integral AUC calculated by IC_50_ was increased gradually as the antiviral activity was improved by the FLJ-FF herb couple knocked in forsythoside A gradually, showing a strong positive correlation, which all indicated that IC_50_ as the weight coefficient was more reasonable than AUC as the weight coefficient.

### 2.3. Effect of COS on the Integral Absorptions of Multiple Caffeic Acid Derivatives in the FLJ-FF Herb Couple Preparations

#### 2.3.1. In Vitro Caco-2 Cell Model

MTT test showed that the FLJ-FF herb couple preparations with or without COS at the studied concentrations were all safe for the Caco-2 cells, and we studied the absorption for four preparations (A, B, C and D) diluted 500 fold with or without COS.

As shown in Figure S2, all of the efflux ratios of caffeic acid derivatives in the FLJ-FF herb couple preparations were approximately 1.0, but the *P*_app_-values (AP-BL) were still low in four preparations, which indicated that the absorption of caffeic acid derivatives might not be affected by transport directions but mainly restricted by the tight junctions, like their monomers absorption mechanism [2,29,30].

As shown in Figure S3, COS at the low (0.003125%), medium (0.025%), and high (0.1%) concentrations caused a significant concentration-dependent increase in the *P*_app_-value for caffeic acid derivatives compared to the control group (*p* < 0.05). The highest *P*_app_-value for neochlorogenic acid, chlorogenic acid, cryptochlorogenic acid, 3,5-dicaffeoylquinic acid, 3,4-dicaffeoylquinic acid, isoforsythoside, forsythoside A, and forsythoside B increased by 9.33, 9.84, 3.13, 8.66, 5.86, 2.10, 2.18 and 2.43 fold for Shuang-Huang-Lian, 3.08, 2.13, 3.05, 4.28, 2.58, 2.41, 2.05 and 2.33 folds for Yin-Qiao-Jie-Du, 2.11, 3.32, 1.64, 2.88, 3.23, 3.59, 1.77 and 4.69 folds for Fufang Qin-Lan, 3.35, 3.07, 3.09, 6.33, 4.28, 4.06, 3.23 and 3.03 folds for Qin-Re-Jie-Du with addition of 0.1% (*w*/*v*) of COS.

**Figure 6:**
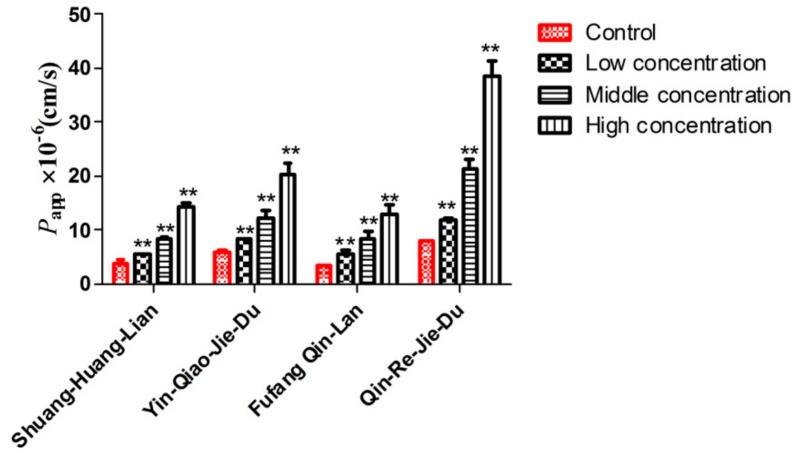
Effect of COS on the integral *P*_app_-value of caffeic acid derivatives in Caco-2 cell in vitro model. (**) *p* < 0.01, compared with control group. (Mean ± SD, *n* = 3).

As shown in Figure 6, the integral *P*_app_-values increased significantly (*p* < 0.01) by 2.67 fold for Shuang-Huang-Lian, 2.56 fold for Yin-Qiao-Jie-Du, 2.96 fold for Fufang Qin-Lan, and 3.92 fold for Qin-Re-Jie-Du, respectively, in the presence of 0.1% (*w*/*v*) of COS.

#### 2.3.2. In Vivo Pharmacokinetic Study

As shown in Figure S4 and Table 3, the AUC value of neochlorogenic acid, chlorogenic acid, cryptochlorogenic acid, 3,5-dicaffeoylquinic acid, 3,4-dicaffeoylquinic acid, isoforsythoside, forsythoside A, and forsythoside B was increased by 6.68, 5.90, 4.17, 3.86, 1.00, 1.98, 0.86 and 2.99 fold for Shuang-Huang-Lian, 0.69, 1.24, 0.80, 1.47, 1.00, 0.52, and 0.86 fold for Yin-Qiao-Jie-Du, 2.72, 1.76, 1.78, 0.54, 1.31, 0.11, 1.07 and 0.32 fold for Fufang Qin-Lan, 0.50, 0.32, 0.74, 1.47, 1.73, 0.22, 0.29 and 0.30 fold for Qin-Re-Jie-Du with the addition of COS at the dosage of 25 mg/kg to rats.

**Figure 7:**
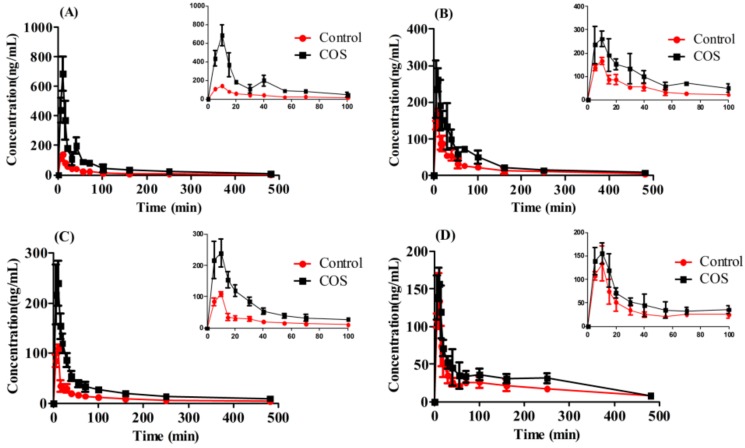
Effect of COS on the integral pharmacokinetic profiles of caffeic acid derivative following oral administration of the FLJ-FF herb couple preparations. ((**A**) Shuang-Huang-Lian extract; (**B**) Yin-Qiao-Jie-Du extract; (**C**) Fufang Qin-Lan extract; (**D**) Qin-Re-Jie-Du extract) (Mean ± SD, *n* = 6).

As shown in Figure 7 and Table 3, the integral AUC-values increased significantly by 2.53 fold for Shuang-Huang-Lian, 0.85 fold for Yin-Qiao-Jie-Du, 1.43 fold for Fufang Qin-Lan, and 0.50 fold for Qin-Re-Jie-Du, respectively.

## 3. Materials

### 3.1. Ethics Statement

The methods were carried out in accordance with the approved guidelines.

All experimental protocols were approved by the Animal Ethics Committee of the Nanjing University of Chinese Medicine (Permit license: SCXK (SU) 2008-0033).

### 3.2. Reagents and Chemicals

Neochlorogenic acid, cryptochlorogenic acid, 3,4-dicaffeoylquinic acid, 3,5-dicaffeoylquinic acid, and forsythoside B (98% pure) were purchased from Sichuan Weikeqi Bio-tech Co., Ltd. (Sichuan, China). Isoforsythoside (98% pure) was purchased from Chengdu Herbpurify Co., Ltd. (Sichuan, China). Chlorogenic acid and forsythoside A were purchased from the National Institute for the Control of Pharmaceutical and Biological Products (Beijing, China). COS (The contents of d-glucosamine, chitosan dimer, chitosan trimer, chitosan tetramer, chitosan pentamer, and chitosan hexamer were 0.42%, 9.19%, 18.78%, 0.69%, 14.48%, and 2.56% of total, respectively) [29] was purchased from Qingdao Honghai Bio-tech Co., Ltd. Formic acid, methanol and acetonitrile (MS grade) were purchased from Merck (Merck, Darmstadt, Germany), and water was purified by a Milli-Q water purification system (Millipore, Bedford, MA, USA). All other chemicals and reagents were of analytical grade.

Dulbecco’s modified Eagle’s medium (DMEM), fetal bovine serum (FBS), 0.05% trypsin-EDTA, penicillin-streptomycin, l-glutamine, and non-essential amino acids were obtained from Gibco BRL Co. (Gaithersburg, MD, USA). Collagen type I, Tosylamide Phenylethyl Chloromethyl Keton-treated Trypsin (trypsin_TPCK), sodium pyruvate, 3-(4,5-dimethylthiazole-2-yl)-2,5-diphenyl tetrazolium bromide (MTT), hank’s balanced salts, phosphate buffered saline (PBS), and DMSO were purchased from Sigma Chemical Co. (St. Louis, MO, USA).

Madin-Darby canine kidney cell lines (MDCK cell, KG067) were purchased from Keygen biotech Co., Ltd. (Nanjing, China). The influenza virus strain, A/PR8/34 (H1N1) was purchased from the Chinese Academy of Preventive Medicine. The human colorectal cancer cell lines (Caco-2, HCT116) were purchased from the cell bank (Chinese Academy of Sciences, Beijing, China).

### 3.3. Plant Material

*Flos Lonicerae Japonicae* (bud of *Lonicera japonica* Thunb., Original place: Henan, China; Voucher number: 111202) and *Fructus forsythiae* (fruit of *Forsythia Suspensa* (Thunb.) Vahl; Original place: Shanxi, China; Voucher number: 111028) were purchased from the Yi-Feng drug store (Nanjing, China) and were authenticated by Prof. Wu (Department of Pharmacognosy, Nanjing University of Chinese medicine). All voucher specimens were deposited in our laboratory for future reference.

## 4. Methods

### 4.1. Study on the Presumption that the Antiviral Effect Enhancement in the Preparations Resulted from the Bioavailability of the FLJ-FF Herb Couple

#### 4.1.1. Preparation of Four Preparations Knocked in or Knocked out FLJ-FF

Shuang-Huang-Lian extract (A) (composition of 1 fold of *Flos Lonicerae Japonicae*, 1 fold of *Fructus Forsythiae*, and 2 folds of *Radix Scutellariae*) was prepared by the procedure described previously [31]. A1 was prepared by A knocked out FLJ-FF herb couple (composition of 4 folds of *Radix Scutellariae*). A2 was prepared by A knocked in FLJ-FF herb couple (composition of 2 folds of *Flos Lonicerae Japonicae*, 2 folds of *Fructus Forsythiae*, and 2 folds of *Radix Scutellariae*). A3 was generated by A1 added COS and A4 was produced by A added COS.

Yin-Qiao-Jie-Du extract (B) (composition of 2.5 folds of *Flos Lonicerae Japonicae*, 2.5 folds of *Fructus Forsythiae*, 1.5 folds of *Herba Menthae Haplocalycis*, 1 folds of *Herba Schizonepetae*, 1.5 folds of *Fructus Arctium*, 1.5 folds of *Radix Platycodi*, 1 fold of *Folium Lophatheri,* and 1.25 folds of *Radix Glycyrrhizae*) was also prepared previously [31]. B1 was prepared by A knocked out FLJ-FF herb couple (composition of 2.5 folds of *Herba Menthae Haplocalycis*, 1.7 folds of *Herba Schizonepetae*, 2.5 folds of *Fructus Arctium*, 2.5 folds of *Radix Platycodi*, 1.6 folds of *Folium Lophatheri,* and 2.1 folds of *Radix Glycyrrhizae*). B2 was prepared by B knocked in FLJ-FF herb couple (composition of 3.6 folds of *Flos Lonicerae Japonicae*, 3.6 folds of *Fructus Forsythiae*, 1.1 folds of *Herba Menthae Haplocalycis*, 0.7 folds of *Herba Schizonepetae*, 1.1 folds of *Fructus Arctium*, 1.1 folds of *Radix Platycodi*, 0.7 fold of *Folium Lophatheri,* and 0.9 folds of *Radix Glycyrrhizae*). B3 was generated by B1 added COS and B4 was produced by B added COS.

Fufang Qin-Lan extract (C) was composed of 1 fold of *Flos Lonicerae Japonicae*, 2 folds of *Fructus Forsythiae*, 1 fold of *Radix Scutellariae* and 1 fold of *Radix Isatidis* [31]. C1 was prepared by C knocked out FLJ-FF herb couple (composition of 2.5 folds of *Radix Scutellariae* and 2.5 folds of *Radix Isatidis*). C2 was prepared by A knocked in FLJ-FF herb couple (composition of 1.25 folds of *Flos Lonicerae Japonicae*, 2.5 folds of *Fructus Forsythiae*, 0.625 folds of *Radix Scutellariae,* and 0.625 folds of *Radix Isatidis*). C3 was generated by C1 added COS and C4 was produced by C added COS.

Qin-Re-Jie-Du extract (D) was composed of 2.48 folds of *Flos Lonicerae Japonicae*, 1.24 folds of *Fructus Forsythiae*, 12.41 folds of *Gypsum Fibrosum*, 1.98 folds of *Radix Scrophulariae*, 1.48 folds of *Radix Rehmanniae*, 1.24 folds of *Fructus Gardeniae*, 1.24 folds of *Radix Scutellariae*, 1.24 folds of *Gentianae*, 1.24 folds of *Radix Isatis*, 1 fold of *Rhizoma Anemarrhenae,* and 1 fold of *Radix Ophiopgonis* [31]. D1 was prepared by D knocked out FLJ-FF herb couple (composition of 14.43 folds of *Gypsum Fibrosum*, 2.30 folds of *Radix Scrophulariae*, 1.72 folds of *Radix Rehmanniae*, 1.44 folds of *Fructus Gardeniae*, 1.44 folds of *Radix Scutellariae*, 1.44 folds of *Gentianae*, 1.44 folds of *Radix Isatis*, 1.16 fold of *Rhizoma Anemarrhenae,* and 1.16 fold of *Radix Ophiopgonis*). D2 was prepared by D knocked in FLJ-FF herb couple (4.35 folds of *Flos Lonicerae Japonicae*, 2.18 folds of *Fructus Forsythiae*, 10.88 folds of *Gypsum Fibrosum*, 1.74 folds of *Radix Scrophulariae*, 1.30 folds of *Radix Rehmanniae*, 1.09 folds of *Fructus Gardeniae*, 1.09 folds of *Radix Scutellariae*, 1.09 folds of *Gentianae*, 1.09 folds of *Radix Isatis*, 0.88 fold of *Rhizoma Anemarrhenae,* and 0.88 fold of *Radix Ophiopgonis*). D3 was generated by D1 added COS and D4 was produced by D added COS.

In brief, the concentration of extracts were 4.0 g raw medicine per milliliter for A, A1, A2, A3 and A4, 8.0 g raw medicine per milliliter for B, B1, B2, B3 and B4, 6.0 g raw medicine per milliliter for C, C1, C2, C3 and C4, and 20.0 g raw medicine per milliliter for C, C1, C2, C3 and C4, respectively. Besides, the concentration of COS in A3, B3, C3, D3, A4, B4, C4 and D4 were all 3.125 mg/mL. The contents of the FLJ-FF couple herb in A, B, C and D (Table S1) were analyzed by our previous method [32].

#### 4.1.2. Effect of Shuang-Huang-Lian Groups (A, A1, A2 and A3), Yin-Qiao-Jie-Du Groups (B, B1, B2 and B3), Fufang Qin-Lan Groups (C, C1, C2 and C3) and Qin-Re-Jie-Du Groups (D, D1, D2 and D3) on the Influenza Virus

Male SD rats (~250 g) were kept in an environmentally controlled breeding room (temperature: 20 ± 2 °C, relative humidity: 60% ± 5%) for 1 week. The rats were fasted for 12 h, and then administrated Shuang-Huang-Lian groups (A, A1, A2 and A3), Yin-Qiao-Jie-Du groups (B, B1, B2 and B3), Fufang Qin-Lan groups (C, C1, C2 and C3) and Qin-Re-Jie-Du groups (D, D1, D2 and D3) at the dosage of 32 g raw medicine per kilogram, 64 g raw medicine per kilogram, 48 g raw medicine per kilogram, and 160 g raw medicine per kilogram, respectively. Whole blood was collected after dosing for 0, 5, 10, 15, 20, 30, 40, 55, 70, 100, 160, 250 and 480 min. After clotted by leaving it undisturbed at room temperature for 30 min, Serum was prepared by centrifuging at 1000× *g* for 10 min in a refrigerated centrifuge, and immediately stored at −70 °C for further analysis after being inactivated by heating to 56°C for 30 min in a water-bath.

#### 4.1.3. Cell Culture and Influenza A Virus Propagation

MDCK cells were grown in DMEM containing 10% FBS, 1% Pen/Strep at 37°C and 5% CO_2_ in a humidified incubator. H1N1 virus was serially diluted 10-fold in DMEM to infect MDCK cells in 96-well plates. Influenza virus infection was determined 48 h post infection using cytopathic effect (CPE) inhibition assays. Virus titer was determination using the Reed-Muench method, and expressed as tissue culture infective dose 50% (TCID_50_).

#### 4.1.4. Cell Viability Assay (Toxicity)

The MTT assay was used to examine the effect of the drug-containing serum on cell viability described previously [12]. MDCK cells in 96-well plates were treated with sequential dilutions or blank serum in a total of 100 μL growth medium for 48 h. Thereafter, 10 μL of 5 mg/mL MTT solution in HBSS was added to each well and the plate was incubated for another 4 h. The solutions in each well were then removed followed by dissolving the remaining formazan crystals in the cells with 200 μL of DMSO. The absorbance of the mixture in the 96-well plate was then measured with a Kinetic microplate reader (Molecular Devices) at 570 nm. The cytotoxicity of drug-containing serum was calculated as the percentage of the absorbance relative to that of the negative control.

#### 4.1.5. Drug-Containing Serum Anti-Viral Effect

MDCK cells seeded in 96-well plate were exposed to influenza A virus (100 TCID_50_/100 μL). After 2 h adsorption in a humidified incubator, the nonadsorbed virus was removed. Subsequently, Serum in a non-cytotoxic concentration was added to MDCK cells to co-culture until CEP reaching 100% for the virus group. Thereafter, an MTT assay was measured for cell viability to evaluate the viral inhibition effect.

### 4.2. Study on the Integral Pharmacokinetics of Caffeic Acid Derivatives Calculated by the Efficacy-Weighting Approach and the AUC-Weighting Method

#### 4.2.1. The Integral Pharmacokinetics Calculated by the Efficacy-Weighting Approach

MDCK cells seeded in 96-well plate were exposed to influenza A virus (100 TCID_50_/100 μL). After 2 h adsorption in a humidified incubator, the nonadsorbed virus was removed. Subsequently, Caffeic acid derivatives of different concentrations (0.0625 mM, 0.1250 mM, 0.2500 mM, 0.5000 mM, and 1.0000 mM) were added to the MDCK cells to co-culture until the CEP reached 100% for virus group. Thereafter, an MTT assay was measured for cell viability to evaluate the viral inhibition effect.

Integral concentrations at each time point for the eight components were calculated based on the anti-viral activity approach. The weighting coefficient for each component was calculated using Equations (1) and (2). The integral concentrations were then calculated by Equation (3), where *w* represents the weighting coefficient and the C_1_–C_8_ represents the plasma concentration of the eight caffeic acid derivatives studied. Half maximal inhibitory concentration (IC_50_) was determined using GraphPad Prism for Windows, version 5 (Graph Pad Software Inc., San Diego, CA, USA).

(1) $$\sum_{1}^{n}\frac{1}{\mathrm{IC}50}=\frac{1}{\mathrm{IC}50_{1}}+\frac{1}{\mathrm{IC}50_{2}}+\frac{1}{\mathrm{IC}50_{3}}+\ldots \ldots +\frac{1}{\mathrm{IC}50_{n}}$$

(2) $$\omega j=\frac{\frac{1}{\mathrm{IC}50_{j}}}{\sum_{1}^{n}\frac{1}{\mathrm{IC}50}}$$

(3) $$CT=\omega_{1}\times C_{1}+\omega_{2}\times C_{2}+\omega_{3}\times C_{3}+\ldots \ldots +\omega_{n}\times C_{n}$$

#### 4.2.2. The Integral Pharmacokinetics Calculated by the AUC-Weighting Method

The pharmacokinetics of caffeic acid derivatives in the FLJ-FF herb couple was studied in our previous study [23].

Integral concentrations at each time point for the eight components were calculated based on the AUC-weighting method. The weighting coefficient for each component was calculated using Equations (4) and (5). The integral concentrations were then calculated by Equation (1), where *w* represents the weighting coefficient and the C_1_–C_8_ represents the plasma concentration of the eight caffeic acid derivatives studied. AUC was determined using GraphPad Prism for Windows, version 5 (Graph Pad Software Inc., San Diego, CA, USA).

(4) $$\sum_{1}^{n}\mathrm{AUC}={\mathrm{AUC}}_{1}+{\mathrm{AUC}}_{2}+{\mathrm{AUC}}_{3}+......+{\mathrm{AUC}}_{n}$$

(5) $$\omega j=\frac{{\mathrm{AUC}}_{i}}{\sum_{1}^{n}\mathrm{AUC}}$$

#### 4.2.3. Rational Evaluation of Integral Pharmacokinetics Calculated by Both IC_50_ and AUC Methods

The rats were administrated the FLJ-FF couple herb as control group [23] and the FLJ-FF couple herb knocked in forsythoside A as the experimental group with a concentration of 25 g raw medicine per kilogram. After dosing for 0, 5, 10, 20, 30, 40, 55, 70, 100, 160, 250, 600 and 1440 min, approximately 300 μL blood was collected from the pre-intubated catheter and put into heparinized 1.5 mL polythene tubes with ascorbic acid (2 μg) at predetermined time points. Subsequently, plasma was prepared by centrifugation at 1816× *g* for 7 min and immediately analyzed or stored at −70 °C for further analysis after being mixed with 10 μL of 5% formic acid.

The treatment and UPLC-ESI-MS/MS analysis for samples have been studied previously [22].

### 4.3. Study on the Effect of COS on the Integral Absorption of Caffeic Acid Derivatives in Preparations for Both In Vitro and In Vivo

#### 4.3.1. In Vitro Caco-2 Cell Model

The protocol of Caco-2 cell study was shown previously [12].

#### 4.3.2. In Vivo Pharmacokinetic Model

The rats were administrated product A and A4 at the dosages of at the dosage of 32 g raw medicine per kilogram, product B and B4 at the dosage of 64 g raw medicine per kilogram, product C and C4 at the dosage of 48 g raw medicine per kilogram, and product D and D4 at the dosage of 160 g raw medicine per kilogram, respectively. After dosing for 0, 5, 10, 15, 20, 30, 40, 55, 70, 100, 160, 250, and 480 min, 300 μL blood was collected from the pre-intubated catheter and put into tubes with heparin sodium injection (10 μL) and ascorbic acid (2 μg) at predetermined time points. Subsequently, plasma was prepared by centrifugation at 1816× *g* for 7 min and immediately analyzed or stored at −70 °C for further analysis after being mixed with 10 μL of 5% formic acid.

#### 4.3.3. Sample Analysis from in Vitro and in Vivo

The treatment and UPLC-ESI-MS/MS analysis for samples collected from in vitro and in vivo models, respectively have been studied previously [23].

## 5. Discussions

TCM has been widely claimed to act in a holistic mode of multiple components and multiple targets. It is necessary to simultaneously characterize multiple representative components for the integrated pharmacokinetics studies of TCM. The integral pharmacokinetics based on an AUC-weighting approach has been successfully applied to the same type constituents derived from TCM [24,25,26,27,28]. Table 1 and Table 2 showed that the antiviral activity of chlorogenic acids among caffeic acid derivatives in the FLJ-FF herb couple was lowest, but the AUC was highest. For example, The AUC of chlorogenic acid in the FLJ-FF herb couple was 42.3 times higher than that of forsythoside A, but antiviral activity was 2.47 folds lower than that of forsythoside A, which meant that the effect of the bioavailability fluctuation of phenylethanoid glycosides on the integral AUC calculated by an AUC-weighting approach was far less than that of chlorogenic acids. Thus, we found (Figure 5A) that the integral AUC in the FLJ-FF herb couple knocked in forsythoside A as the experimental group was not significant, compared with that in the FLJ-FF herb couple as the control group. However, Figure 5C showed that the antiviral activity was improved significantly as forsythoside A knocked in the FLJ-FF herb couple, which indicated that an AUC-weighting method might not be suitable for studying integral pharmacokinetics of caffeic acid derivatives. Surprisingly, Figure 5B showed that the integral AUC calculated by IC_50_ as weight coefficient index was improved significantly as the FLJ-FF herb couple was knocked in forsythoside A, and increased gradually as the antiviral activity was improved by knocked in forsythoside A gradually in the FLJ-FF herb couple, showing a strong positive correlation (Figure 5D). Besides, the integral pharmacokinetics parameters using IC_50_ as the weight coefficient index, such as *T*_1/2_, AUC, *C*_max_, and *MRT* calculated by DAS 2.1.1 could fully take eight caffeic acid derivatives’ pharmacokinetics parameters into account (Table S2 and Figure S5). The results above indicated that IC_50_ as the weight coefficient was more reasonable than AUC as the weight coefficient.

In order to illustrate the rationality of antiviral activity of the FLJ-FF herb couple preparations improved by COS, it was shown that the enhancement of the antiviral effect for four preparations attributed to the improvement of bioavailability for the FLJ-FF herb couple in vivo (Figure 3). We also found that caffeic acid derivatives were the most important type of active components for antiviral activity in the FLJ-FF herb couple by both PK-PD and DDI models previously [18,23], but had different antiviral effects. In order to better elucidate their contribution to total efficacy, an integral pharmacokinetics based on IC_50_ as the weight coefficient index was established, and found that the integral absorption of caffeic acid derivatives in four preparations can be improved significantly both in vitro and in vivo by COS, which was consistent with the results that the absorption of caffeic acid derivatives in the FLJ-FF herb couple preparations mainly restricted by the tight junctions (Figures S2 and S3), and COS was an absorption enhancer based on tight junctions with high effectiveness and low mucosal toxicity [11].

## 6. Conclusions

All findings above suggested that the enhancement of antiviral effect for the FLJ-FF herb couple preparations by COS resulted from the integral AUC improvement for caffeic acid derivatives, and COS can be considered as a promising absorption enhancer to improve the efficacy of the FLJ-FF herb couple preparations especially that of Shuang-Huang-Lian.

## Figures and Tables

**Table 1:** Weighting factor based on anti-viral activity-IC_50_.

Parameters	Neochlorogenic Acid	Chlorogenic Acid	Cryptochlorogenic Acid	3,5-Dicaffeoylquinic Acid	3,4-Dicaffeoylquinic Acid	Isoforsythoside	Forsythoside A	Forsythoside B
IC_50_	0.722	0.754	0.735	0.393	0.401	0.403	0.305	0.360
Wj	0.0786	0.0753	0.0772	0.144	0.140	0.141	0.186	0.158

Mean values performed by W.Z. and A.Y.

**Table 2:** Weighting factor based on AUC.

Parameters	Neochlorogenic Acid	Chlorogenic Acid	Cryptochlorogenic Acid	3,5-Dicaffeoylquinic Acid	3,4-Dicaffeoylquinic Acid	Isoforsythoside	Forsythoside A	Forsythoside B
AUC	114,687.70	523,507.70	141,043.20	8996.03	6227.13	10,128.24	12,648.30	6871.71
Wj	0.139	0.635	0.171	0.011	0.007	0.012	0.015	0.008

**Table 3:** Effects of COS on the oral bioavailability of caffeic acid derivatives in the FLJ-FF herb couple preparations (Mean ± SD, *n* = 6).

Components	Shuang-Huang-Lian Oral Liquid			Yin-Qiao-Jie-Du Oral Liquid			Fufang Qin-Lan Oral Liquid			Qin-Re-Jie-Du Oral Liquid		
Caffeic Acid Derivatives	AUC (Control)	AUC (Absorption Enhancer)	Ratio	AUC (Control)	AUC (Absorption Enhancer)	Ratio	AUC (Control)	AUC (Absorption Enhancer)	Ratio	AUC (Control)	AUC (Absorption Enhancer)	Ratio
Neochlorogenic acid	10,646 ± 1009.8	81,789 ± 9230.3 **	7.6829	16,217 ± 2946.9	27,324 ± 3763.5 **	1.6850	7706.5 ± 1066.0	28,664 ± 2028.7 **	3.7195	16,826 ± 4489.8	25,208 ± 2157.4 *	1.4982
Chlorogenic acid	38,279 ± 5334.8	26,428 × 10 ± 40,480 **	6.9041	43,537 ± 5549.5	97,656 ± 16040 **	2.2431	22,643 ± 4304.1	62,547 ± 3675.1 **	2.7623	44,095 ± 14,327	58,100 ± 2892.1 *	1.3176
Cryptochlorogenic acid	14,240 ± 1448.9	73,548 ± 7090.8 **	5.1648	17,123 ± 1294.8	30,770 ± 6347.8 **	1.7970	6771.0 ± 1078.0	18,832 ± 2037.5 **	2.7813	23,515 ± 4239.6	40,972 ± 754.48 **	1.7423
3,5-dicaffeoylquinic acid	3460.5 ± 260.90	16,812 ± 3170.7 *	4.8583	1730.3 ± 28.868	4278.5 ± 686.84 **	2.4728	1889.6 ± 324.64	2911.3 ± 359.13 *	1.5407	2617.8 ± 697.59	6460.0 ± 968.28 **	2.4678
3,4-dicaffeoylquinic acid	814.80 ± 84.113	1627.5 ± 9.1924 *	1.9974	1383.0 ± 114.56	2769.8 ± 13.435 **	2.0027	847.53 ± 151.80	1955.8 ± 210.57 **	2.3077	480.73 ± 54.438	1313.0 ± 192.84 **	2.7313
Isoforsythoside	2711.0 ± 226.46	8080.0 ± 941.16 **	2.9805	2012.2 ± 65.200	4343.3 ± 313.96 *	2.1585	2393.8 ± 129.40	2667.0 ± 156.62	1.1142	5516.7 ± 537.39	6750.7 ± 292.74 *	1.2237
Forsythoside A	11,776 ± 272.44	21,906 ± 369.82 *	1.8602	15,629 ± 2641.4	23,727 ± 1224.0 *	1.5182	5581.0 ± 591.26	11,555 ± 1131.1 **	2.0703	10,438 ± 1181.5	13,446 ± 354.66 **	1.2882
Forsythoside B	3685.0 ± 328.86	14,694 ± 2047.0 **	3.9875	6678.3 ± 850.79	12,440 ± 1564.9 **	1.8628	1416.5 ± 252.44	1868.2 ± 142.50 *	1.3189	1955.0 ± 716.78	2532.0 ± 183.95	1.2951
Integration	7294.0 ± 613.83	25,748 ± 355.67 **	3.5300	9139.0 ± 1668.6	16,865 ± 1202.1 *	1.8454	5283.0 ± 653.58	12,811 ± 2545.9 **	2.4249	9904.0 ± 886.13	14,818 ± 2331.3 *	1.4962

(*) *p* < 0.05 and (**) *p* < 0.01, compared with control group.

## Supplementary Materials

Supplementary materials are available online.

## References

[1] Shang X., Pan H., Li M., Miao X., Ding H. (2011). *Lonicera japonica* Thunb.: Ethnopharmacology, phytochemistry and pharmacology of an important traditional Chinese medicine. J. Ethnopharmacol..

[2] Zhou W., Qin K.M., Shan J.J., Ju W.Z., Liu S.J., Cai B.C., Di L.Q. (2012). Improvement of intestinal absorption of forsythoside A in weeping forsythia extract by various absorption enhancers based on tight junctions. Phytomedicine.

[3] Lin L.M. (2008). The Methods of Efficient Components Recognition-Flos Lonicerae Japonicae-Fructus Forsythiae Herb Couple as a Typical Example. Ph.D. Thesis.

[4] He W.Y., Gao R.M., Li X.Q., Jiang J.D., Li Y.H. (2010). In vitro anti-influenza virus activity of 10 traditional Chinese medicines. Yao Xue Xue Bao.

[5] Yu J.S., Ho C.H., Hsu Y.C., Wang J.J., Hsieh C.L. (2014). Traditional Chinese medicine treatments for upper respiratory tract infections/common colds in Taiwan. Eur. J. Integr. Med..

[6] Zhang H., Chen Q., Zhou W., Gao S., Lin H., Ye S., Xu Y., Cai J. (2013). Chinese Medicine Injection Shuanghuanglian for Treatment of Acute Upper Respiratory Tract Infection: A Systematic Review of Randomized Controlled Trials. Evid. Based Complement. Altern. Med..

[7] Zhang W.B., Jiang H.L., Zhou W., Zhong Y.Q., Yang H.M., Fu J.J., Mao B. (2009). Chinese medicine for acute upper respiratory tract infection: A systematic review of randomized controlled trials. Zhong Xi Yi Jie He Xue Bao.

[8] Fang T.H., Xiang Y.H. (2009). Lifang & Partners, Assignee. Compound Qinlan Oral Liquid. China Patent.

[9] Zhou X.M., Lu C.N., Qi W.B., Ma Y.J., Tang Y.Z., Chen J.X. (2011). In vivo anti-avian influenza virus activity of Qingkailing and Shuanghuanglian Orals. Chin. Tradit. Herb. Drugs.

[10] Zu M., Zhou D., Gao L., Liu L., Du G.H. (2010). Evaluation of Chinese traditional patent medicines against influenza virus in vitro. Yao Xue Xue Bao.

[11] Gao Y., He L., Katsumi H., Sakane T., Fujita T., Yamamoto A. (2013). Improvement of intestinal absorption of insulin and water-soluble macromolecular compounds by chitosan oligomers in rats. Int. J. Pharm..

[12] Zhou W., Wang H.D., Zhu X.X., Shan J.J., Yin A.L., Cai B.C., Di L.Q. (2013). Improvement of Intestinal Absorption of Forsythoside A and Chlorogenic Acid By Different *Carboxymethyl* Chitosan and Chito-oligosaccharide, Application to *Flos Lonicerae*—*Fructus Forsythiae* Herb Couple Preparations. PLoS ONE.

[13] Kumar N., Singh B., Bhandari P., Gupta A.P., Uniyal S.K., Kaul V.K. (2005). Biflavonoids from Lonicera japonica. Phytochemistry.

[14] Ozaki Y., Rui J., Tang Y., Satake M. (1997). Anti-inflammatory effect of Forsythia suspensa Vahl and its active fraction. Biol. Pharm. Bull..

[15] Rouf A.S., Ozaki Y., Rashid M.A., Rui J. (2001). Dammarane derivatives from the dried fruits of Forsythia suspense. Phytochemistry.

[16] Zhou W., Tan X.B., Shan J.J., Wang S.Q., Yin A.L., Cai B.C., Di L.Q. (2014). Study on the main components interaction from *Flos Lonicerae* and *Fructus Forsythiae* and their dissolution in vitro and intestinal absorption in rats. PLoS ONE.

[17] Li H.W. (2011). The Study on Forsythiaside A Inhibit IBV and Regulate the Expression of IFN-α Signaling Pathway-Related Factors. Master’s Thesis.

[18] Qu H., Zhang Y., Wang Y., Li B., Sun W. (2008). Antioxidant and antibacterial activity of two compounds (forsythiaside and forsythin) isolated from forsythia suspense. J. Pharm. Pharmacol..

[19] Wang L. (2010). Study of Effective Substances Screening for Flos Lonicerae Based on the “Spectrum-Effect” Combination. Master’s Thesis.

[20] Wu P.P., Yan M., Huo S.X. (2011). Research progress on phenylethanoid glycosides. Herald Med..

[21] Zhang L.W. (2002). Extraction Method and Biological Activities of Forsythiaside. Master’s Thesis.

[22] Zhang T.T. (2011). Novel Patterns of Efficient Components Recognition and Quality Control for Flos Lonicerae Japonicae Based on Constituent Knock-Out/Knock-In. Master’s Thesis.

[23] Zhou W., Tam K.Y., Meng M.X., Shan J.J., Wang S.Q., Ju W.Z., Cai B.C., Di L.Q. (2015). Pharmacokinetics screening for multi-components absorbed in the rat plasma after oral administration of traditional Chinese medicine-Flos Lonicerae Japonicae—Fructus Forsythiae herb couple by sequential negative and positive ionization ultra performance liquid chromatography/tandem triple quadrupole mass spectrometric detection. J. Chromatogr. A.

[24] He W., Liu G., Cai H., Sun X., Hou W., Zhang P., Xie Z., Liao Q. (2014). Integrated pharmacokinetics of five protoberberine-type alkaloids in normal and insomnic rats after single and multiple oral administration of Jiao-Tai-Wan. J. Ethnopharmacol..

[25] Ma Z.T., Yang X.W., Zhang Y., Liu J.X. (2014). Pharmacochemistry and integrated pharmacokinetics of six alkaloids after oral administration of Huang-Lian-Jie-Du-Tang decoction. J. Asian Nat. Prod. Res..

[26] Pan L., Zhou J., Zhu H., Wang W., Zhang M., Tian X., Lu J., Zeng M. (2014). Study on integrated pharmacokinetics of gardenia acid and geniposide: Time-antioxidant efficacy after oral administration of Huanglian-Zhizi couplet medicine from Huang-Lian-Jie-Du-Tang in MCAO rats. Am. J. Chin. Med..

[27] Xie Y., Hao H., Kang A., Liang Y., Xie T., Sun S., Dai C., Zheng X., Xie L., Li J., Wang G. (2010). Integral pharmacokinetics of multiple lignan components in normal, CCl4-induced hepatic injury and hepatoprotective agents pretreated rats and correlations with hepatic injury biomarkers. J. Ethnopharmacol..

[28] Zhu H., Qian Z., Li H., Guo L., Pan L., Zhang Q., Tang Y. (2012). Integrated pharmacokinetics of major bioactive components in MCAO rats after oral administration of Huang-Lian-Jie-Du-Tang. J. Ethnopharmacol..

[29] Zhou W., Tan X.B., Shan J.J., Liu T., Cai B.C., Di L.Q. (2014). Effect of chito-oligosaccharide on the intestinal absorptions of phenylethanoid glycosides in Fructus Forsythiae extract. Phytomedicine.

[30] Zhou W., Shan J.J., Wang S.C., Cai B.C., Di L.Q. (2015). Transepithelial transport of phenolic acids in Flos Lonicerae Japonicae in intestinal Caco-2 cell monolayers. Food Funct..

[31] Chinese Pharmacopoeia Commission (2015). The Pharmacopoeia of the People’s Republic of China Version.

[32] Zhou W., Shan J.J., Ju W.Z., Wang S.C., Meng M.X., Cai B.C., Di L.Q. (2015). Simultaneous determination of twenty-six components of Flos Lonicerae Japonicae—Fructus Forsythiae herb couple using UPLC-ESI-MS/MS, Application to its preparations. Anal. Methods.

