# Supplementary material for: Study on the Rationality for Antiviral Activity of Flos Lonicerae Japonicae-Fructus Forsythiae Herb Couple Preparations Improved by Chito-Oligosaccharide via Integral Pharmacokinetics
Source: Molecules. 2017 Apr 20;22(4):654. doi: 10.3390/molecules22040654 (PMC6154603; doi:10.3390/molecules22040654)
Supplement: Supplementary file 1 [file molecules-22-00654-s001.pdf]

Supporting Information

Study on Rationality for Antiviral Activity of *Flos Lonicerae Japonicae-Fructus Forsythiae* Herb Couple Preparations iIproved by Chito-Oligosaccharide via Integral Pharmacokinetics

Wei Zhou, Ailing Yin, Jinjun Shan, Shouchuan Wang, Baochang Cai, Liuqing Di*

* Corresponding author. Tel.: +86 25 86798226; fax: +86 25 83271038

E-mail address: diliuqing@hotmail.com

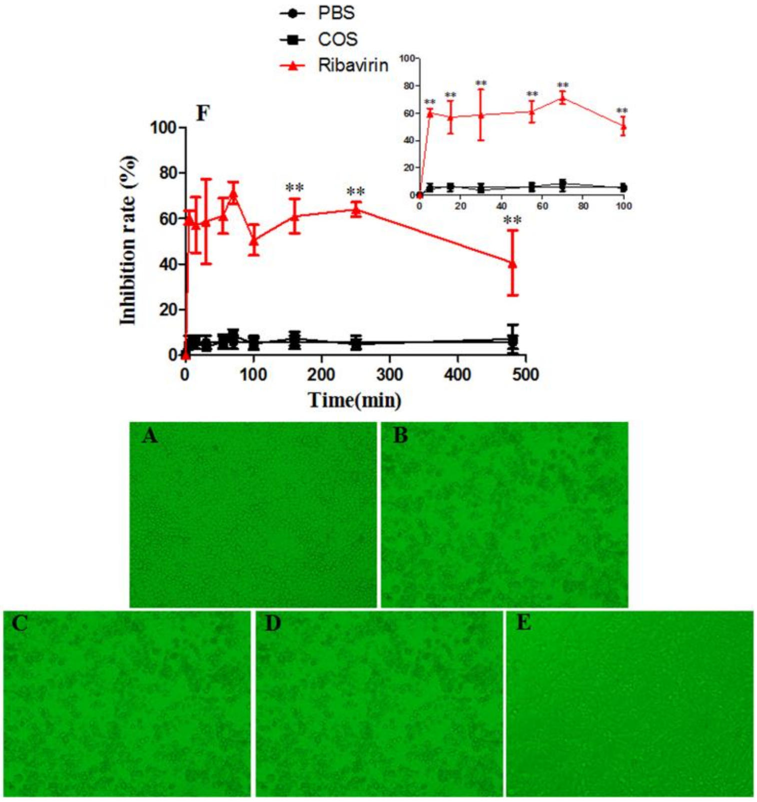

Supplementary Fig. S1 Cytopathogenic effect observed on influenza virus infections in MDCK cells (A: Normal group; B: Virus group; C: PBS group; D: COS group; E: Ribavirin as positive control group; F: Inhibition rate of COS and ribavirin on influenza virus); Inhibition rate was assayed with MTT and expressed as percentage of controls (data ±S.D. *n*=8). (*) *P*<0.05 and (**) *P*<0.01, compared with PBS group.

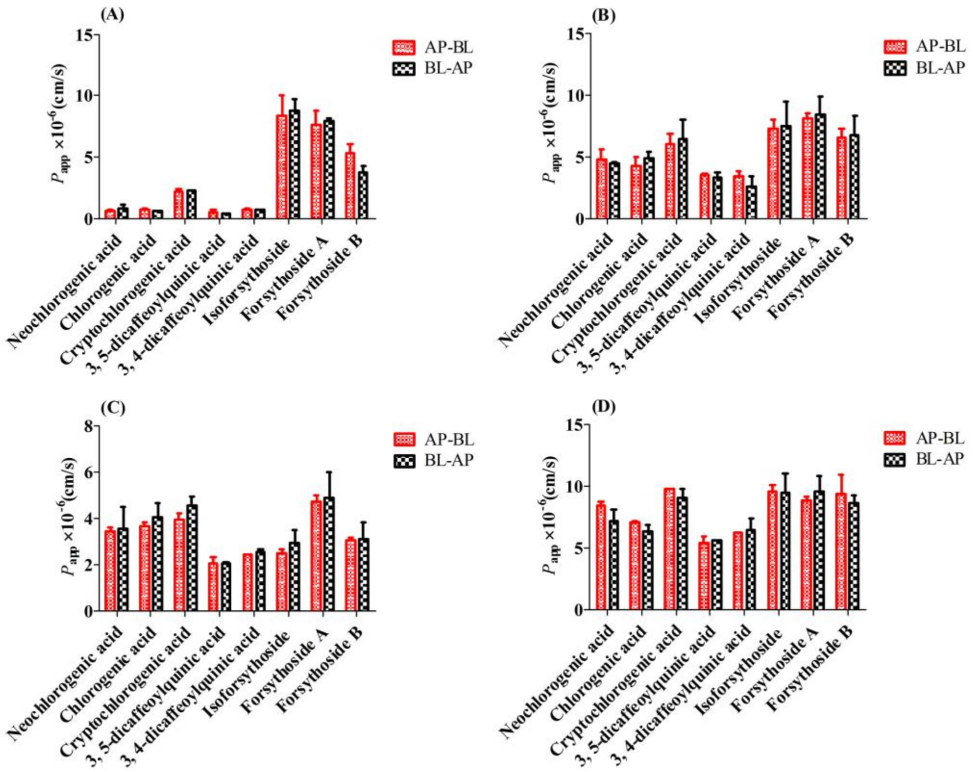

Supplementary Fig. S2 Bidirectional permeation of caffeic acid derivatives in the FLJ-FF herb couple preparations across Caco-2 cell monolayers. (A: Shuang-Huang-Lian extract; B: Yin-Qiao-Jie-Du extract; C: Fufang Qin-Lan extract; D: Qin-Re-Jie-Du extract)

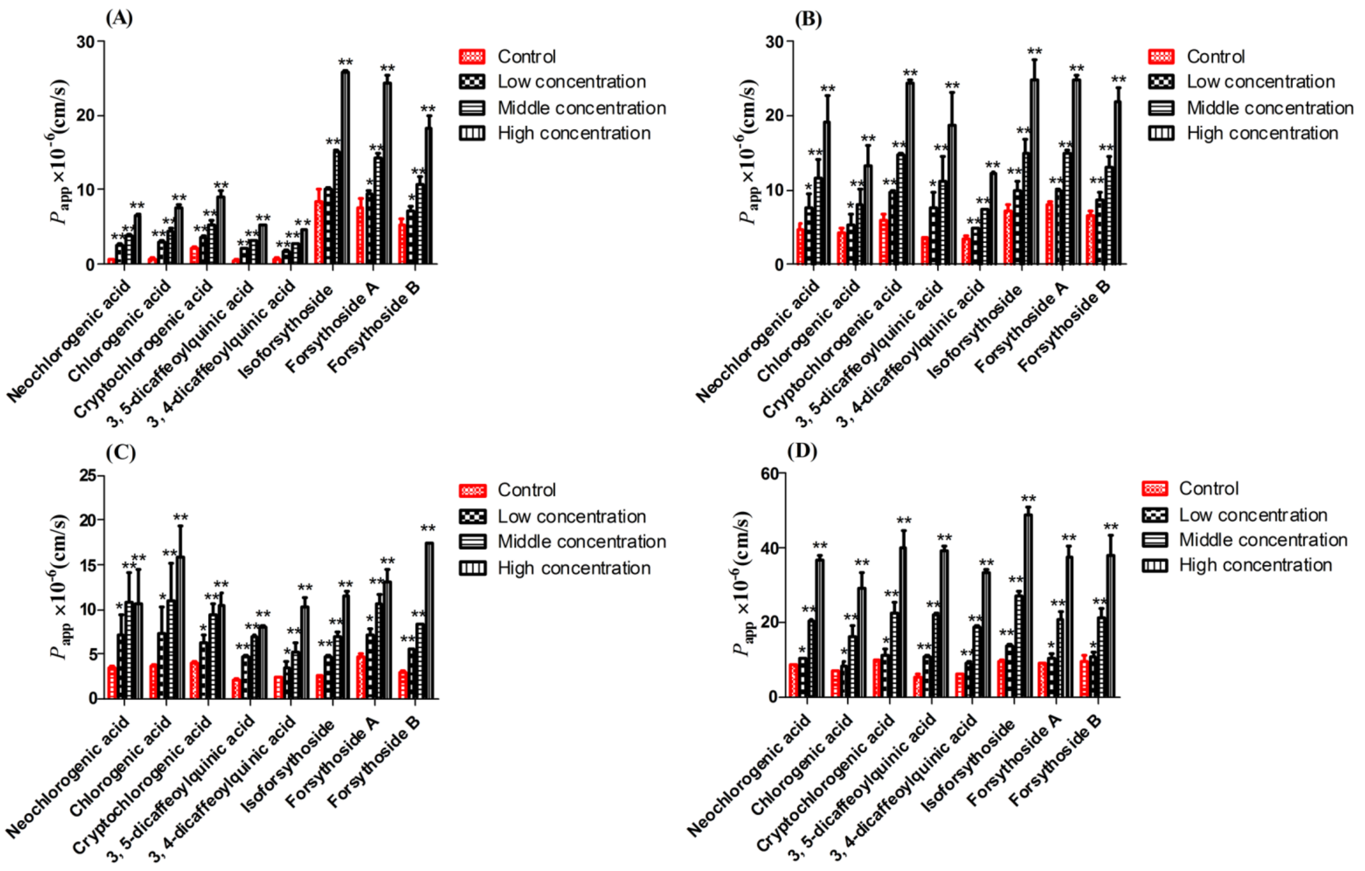

Supplementary Fig. S3 Effect of COS on P_{app}-value of caffeic acid derivatives in Caco-2 cell *in vitro* model. Results are expressed as the mean ± S.D. (*) P<0.05 and (**) P<0.01 compared with the control group. (A: Shuang-Huang-Lian extract; B: Yin-Qiao-Jie-Du extract; C: Fufang Qin-Lan extract; D: Qin-Re-Jie-Du extract)

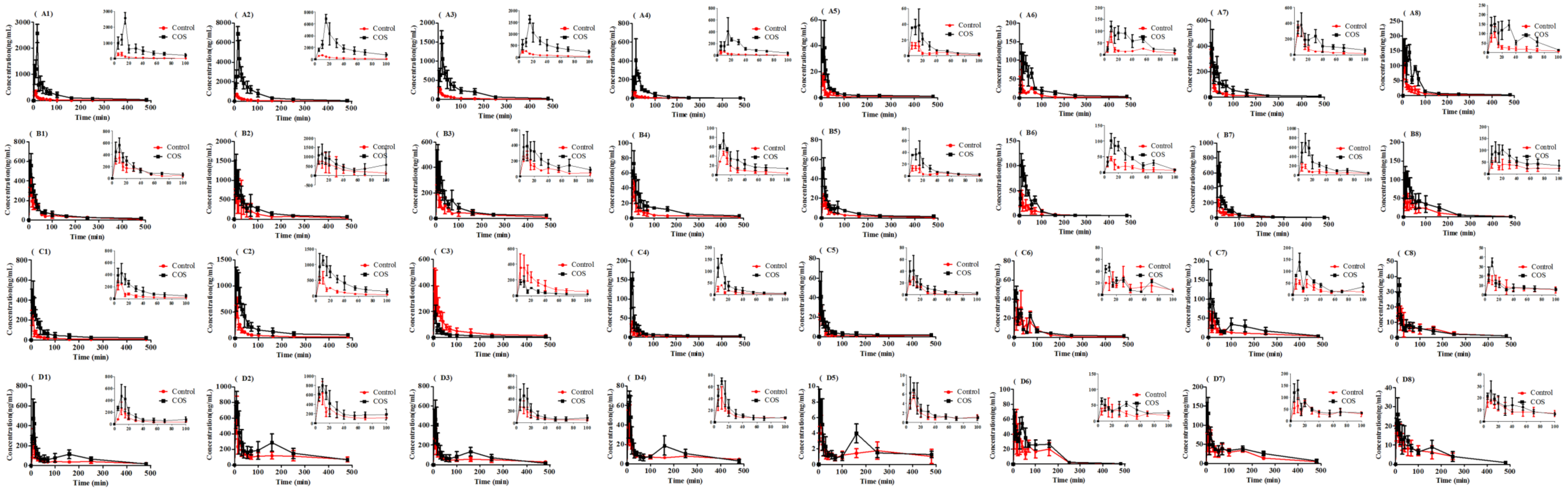

Supplementary Fig. S4 Effect of COS on the mean pharmacokinetic profiles of caffeic acid derivative following oral administration of FLJ-FF herb couple preparations. (Ingredients 1, 2, 3, 4, 5, 6, 7, and 8 represent neochlorogenic acid, chlorogenic acid, cryptochlorogenic acid, 3, 5-dicaffeoylquinic acid, 3, 4-dicaffeoylquinic acid, isoforsythoside, forsythoside A, and forsythoside B, respectively; A, B, C, and D represent Shuang-Huang-Lian extract, Yin-Qiao-Jie-Du extract, Fufang Qin-Lan extract, and Qin-Re-Jie-Du extract)

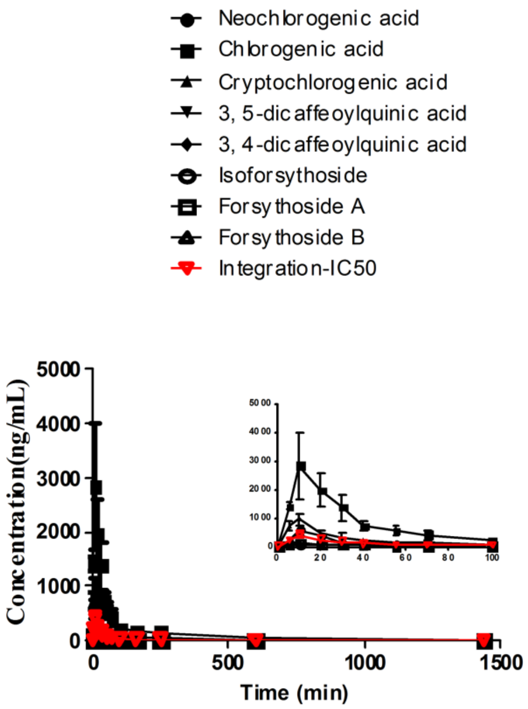

Supplementary Fig. S5 Integrated pharmacokinetic profiles of caffeic acid derivatives following oral administration of the FLJ-FF herb couple based on IC50.

Supplementary Table S1 The contents of the FLJ-FF herb couple in four preparations (μg/mL)

No.	Compounds	Shuang-Huang-Lian（A）	Yin-Qiao-Jie-Du（B）	Fufang Qin-Lan（C）	Qin-Re-Jie-Du（D）
01	Forsythoside B	394.60	521.40	454.60	583.90
02	Loganin	129.00	206.00	245.10	284.00
03	Macranthoidin B	8.32	3.83	12.15	29.95
04	Dipsacoside B	4.89	3.98	8.12	23.85
05	Rutin	448.30	503.60	411.20	594.90
06	Arctiin	35.78	2561.00	71.90	2029.00
07	Phillyrin	1019.00	703.60	1455.00	1741.00
08	Pinoresinol-β-D-glucoside	683.50	782.10	739.20	1243.00
09	3, 5-dicaffeoylquinic acid	903.00	1065.00	882.70	1244.00
10	3, 4-dicaffeoylquinic acid	1416.00	1753.00	1278.00	1916.00
11	Isoquercitrin	275.20	335.70	318.80	389.90
12	Hyperoside	604.20	677.70	626.30	934.80
13	Astragalin	90.53	119.10	142.20	198.50
14	Luteoloside	1600.00	1746.00	1656.00	1706.00
15	Genistin	2.59	4.07	10.74	6.26
16	Arctigenin	11.56	105.90	15.38	69.19
17	Neochlorogenic acid	1782.00	2282.00	1644.00	3449.00
18	Chlorogenic acid	5995.00	6809.00	5109.00	7477.00
19	Cryptochlorogenic acid	1747.00	2125.00	1986.00	3123.00
20	Quercetin	60.95	43.72	28.51	71.51
21	Luteolin	24.39	26.60	28.15	38.83
22	Genistein	14.43	3.96	2.50	6.49
23	Quinic acid	8995.00	10090.00	9689.00	11950.00
24	Caffeic acid	90.72	134.40	127.00	309.60
25	Isoforsythoside	195.40	374.60	445.50	1119.00
26	Forsythoside A	2876.00	3210.00	2772.00	4602.00

Supplementary Table S2 Integrated pharmacokinetic parameters of caffeic acid derivatives in the FLJ-FF herb couple based on IC50

Parameters	C_{max} (ng·mL^{-1})	T_{max} (min)	$AUC_{0\text{-}t}$ (ng·min·mL^{-1})	$AUC_{0\text{-}\infty}$ (ng·min·mL^{-1})	$MRT_{0\text{-}t}$ (min)	$T_{1/2z}$ (min)
**Neochlorogenic acid**	575.38±170.08	10.00±0.00	34729.00±3634.50	42909.00±11047.00	317.90±54.37	209.65±25.37
**Chlorogenic acid**	2498.30±437.76	10.00±0.00	193470.00±58626.00	189670.00±62359.00	284.35±60.95	234.22±41.92
**Cryptochlorogenic acid**	949.78±201.21	10.00±0.00	42751.00±10512.00	43586.00±10547.00	290.35±66.55	278.21±61.53
**3, 5-dicaffeoylquinic acid**	70.50±28.05	10.00±0.00	3188.00±625.91	3250.50±650.12	383.41±31.29	356.85±33.56
**3, 4-dicaffeoylquinic acid**	66.74±15.36	10.00±0.00	8451.90±892.70	12802.00±1084.70	455.94±30.63	551.05±38.97
**Isoforsythoside**	104.55±15.78	10.00±0.00	5631.60±554.83	5751.50±490.20	293.12±84.17	234.12±12.32
**Forsythoside A**	115.04±32.06	10.00±0.00	5909.50±491.96	6224.50±374.57	247.26±37.61	251.96±41.35
**Forsythoside B**	51.96±14.52	10.00±0.00	4292.70±153.20	4315.40±183.84	202.10±57.04	245.33±55.52
**Integration-IC50**	396.22±133.59	10.00±0.00	25176.00±1629.00	25926.00±6790.80	302.38±51.27	296.94±39.00
